# Probing structural changes in single enveloped virus particles using nano-infrared spectroscopic imaging

**DOI:** 10.1371/journal.pone.0199112

**Published:** 2018-06-12

**Authors:** Sampath Gamage, Marquez Howard, Hiroki Makita, Brendan Cross, Gary Hastings, Ming Luo, Yohannes Abate

**Affiliations:** 1 Department of Physics and Astronomy, University of Georgia, Athens, Georgia, United States of America; 2 Department of Physics and Astronomy, Georgia State University, Atlanta, Georgia, United States of America; 3 Department of Chemistry, Georgia State University, Atlanta, Georgia, United States of America; Centre de Recherche en Cancerologie de Lyon, FRANCE

## Abstract

Enveloped viruses, such as HIV, Ebola and Influenza, are among the most deadly known viruses. Cellular membrane penetration of enveloped viruses is a critical step in the cascade of events that lead to entry into the host cell. Conventional ensemble fusion assays rely on collective responses to membrane fusion events, and do not allow direct and quantitative studies of the subtle and intricate fusion details. Such details are accessible via single particle investigation techniques, however. Here, we implement nano-infrared spectroscopic imaging to investigate the chemical and structural modifications that occur prior to membrane fusion in the single archetypal enveloped virus, influenza X31. We traced in real-space structural and spectroscopic alterations that occur during environmental pH variations in single virus particles. In addition, using nanospectroscopic imaging we quantified the effectiveness of an antiviral compound in stopping viral membrane disruption (a novel mechanism for inhibiting viral entry into cells) during environmental pH variations.

## Introduction

Many enveloped viruses continue to be a persistent health threat to human populations. To enter a cell, viruses attach to host-cell receptors. Detailed understanding of how viral entry proteins interact with their host-cell receptors and how the viral membrane envelope undergoes changes that lead to entry offer opportunities for the development of novel therapeutics and vaccines. For example, influenza virus (IFV) has been used as a prototype enveloped virus to study virus entry into the host cell. Hemagglutinin (HA) is a major surface glycoprotein embedded in the IFV membrane envelope. HA is responsible for IFV attachment to the host cell receptor and is involved in mediating membrane fusion during virus entry. Previous studies have led to a generally accepted model for the fusion mechanism between the target and viral membranes [1]. In this model, the postulated role of HA is primarily to bring the two membranes close to each other, so that they fuse forming a fusion pore. This process was proposed to be initiated by the conformational change of HA induced by low pH. The HA fusion peptide is released and then inserted in the target membrane as the result of this conformation change. Afterward, the trimeric coiled coil of HA refolds to bring the target and viral membranes together for fusion. The fusion process may consist of steps of hemifusion, pore formation and expansion to allow the release of the viral genome. The key point here is that the pore will only form when membrane fusion occurs. However, other reports have observed “rupture” of the target and viral membranes independently before fusion occurs [2, 3]. When a liposome was incubated with a vesicle that has HA on its surface, the low pH induced structural change of HA actually caused rupture in the liposomal membrane (forming gaps) prior to HA refolding [2]. Similar disruption of the cellular membrane was also observed when an adenovirus protein was incubated with the host cell [4]. These results clearly demonstrated that the target host membrane may be disrupted for virus entry without membrane fusion. On the other hand, the viral membrane and the target host membrane do not have the same chemical composition or structure. The requirements for pore formation in each respective membrane are different as shown previously [5–7]. When HA is inserted in the viral envelope, it increases the lipid order [5]. Acylation of HA increased the curvature of the viral membrane [6]. These suggest that the requirement for disrupting the viral membrane may be different from that for the cellular membrane. In addition, it is also unclear if disruption of the viral membrane requires membrane fusion. pH-dependent rupture of the vesicle membrane was observed at low pH when the inside of the vesicle was lined with influenza virus protein M1 that is the viral matrix protein bound with the cytoplasmic tail of HA [7]. These data imply that the disruption of the target or the viral membrane may be induced independently.

Detailed understanding the complex sequential fusion mechanism at a single virus level offers a tremendous opportunity to design antiviral compounds tuned to interrupt the cascade of events leading to viral infection. Conventional ensemble fusion assays relay on collective responses of fusion events and as a result cannot allow direct quantification of some of the subtle fusion details that are more accessible via studies of single isolated particles. Appropriate single virus particle interrogation tools that not only offer high spatial resolution but also possess the capability to probe mechanical/chemical properties and effects of environmental changes could facilitate detailed understanding of viral membrane fusion. For example, the viral infection process causes chemical and structural composition alterations of both the viral and the host cellular membranes at the molecular level, and these changes can be probed by molecular-specific infrared spectroscopic techniques. The diffraction limit is a bottleneck for laser based spectroscopy investigations of single viruses due to their nanoscale size. Scattering type scanning near-field optical microscope (s-SNOM) is not limited by diffraction, and provides the ability to undertake (infrared) spectroscopic studies of objects with a spatial resolution down to 10 nm, allowing high resolution in situ spectroscopic chemical and structural identification and mapping of a single virus and its interaction with environmental triggers and a cell. s-SNOM functions as a local probe via scattering of optical fields from a nano-localized probe tip, either at single frequency or using broadband light sources covering the broad infrared (IR) fingerprint frequency range (see Methods). Simultaneously acquired topography, IR amplitude and phase images in s-SNOM offer complementary information. We implemented nano-infrared spectroscopic imaging to investigate the chemical and structural modifications in influenza X31 during interaction with various environmental pH variations. We have also assessed quantitatively the ability of antiviral compound (Compound 136) in halting viral membrane modification required prior to release of viral genome during environmental pH variations.

## Results and discussion

**Fig 1:**
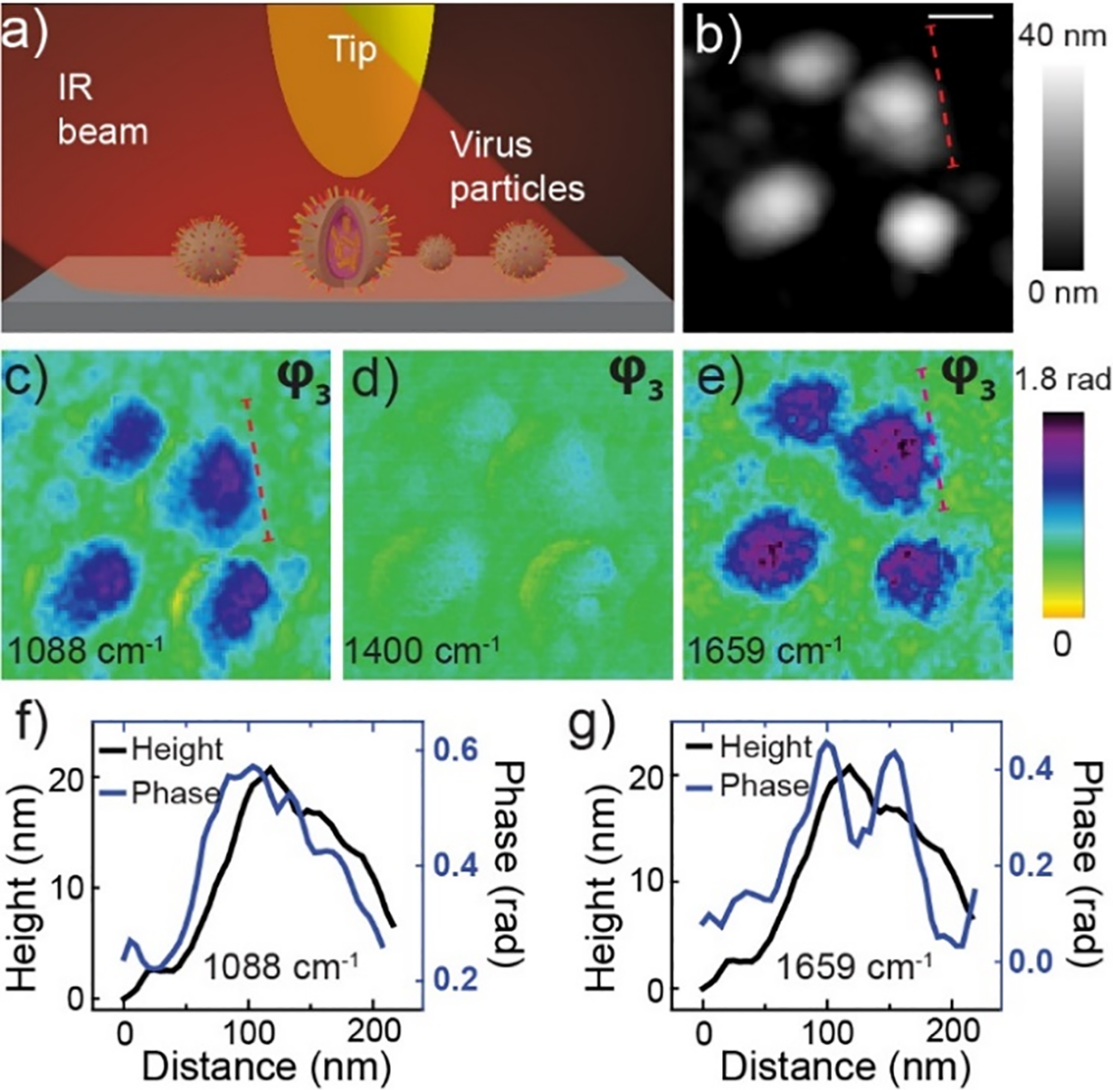
s-SNOM experimental setup and near-field infrared spectral images of influenza virus at pH 7.4. (a) schematic of the s-SNOM experiment, (b) topography of four viruses (scale bar is 100 nm), (c-e) near-field phase (φ_3_) spectral images at three different frequencies. Line profiles of topography (f) and phase (g) representing the red broken lines shown in the topography (b) and phase (c, e) images.

As shown in Fig 1A s-SNOM functions as a nanolocalized probe via scattering of optical fields from a probe tip either at single frequency or using broadband light sources covering a wide IR frequency range. Fig 1B shows a topography image of viruses with height in the range ~20–30 nm (Fig 1F and 1G) and diameter ~70–100 nm. Near-field phase (φ_3_) images are shown at 1088 (Fig 1C), 1400 (Fig 1D) and 1659 cm^-1^ (Fig 1E). It is well established that the near-field phase, being directly proportional to the imaginary part of the permittivity, represents infrared vibrational absorption of a sample [8–10]. The contrast at 1088 and 1659 cm^-1^ indicate strong infrared absorption bands of the virus at these frequencies. The weak contrast at 1400 cm^-1^ demonstrates weak absorption at this frequency. From these near-field spectral images chemical specific structural properties of single viral particles can be assessed [11]. Remarkably, the spectral image at 1659 cm^-1^ (Fig 1E) and the corresponding line profile (Fig 1G) show HA aggregates protruding out from the surface of the virus envelope. These aggregates are not well resolved in the topography map (Fig 1B). We believe these protrusions are HA protein aggregates because they are missing in the 1088 cm^-1^spectral image, which occurs because protein absorbs at 1659 cm^-1^ but not at 1088 cm^-1^. In this way these spectral images provide insight into not only the spatial structure, but also the chemical composition of the surface of the virus. The near-field amplitude images also can distinctly identify the HA protein from the inner structure of the virus (S1 Fig). However, detailed assignment of vibrational modes requires detailed spectra over a broad range, and it is to this issue that we now turn.

**Fig 2:**
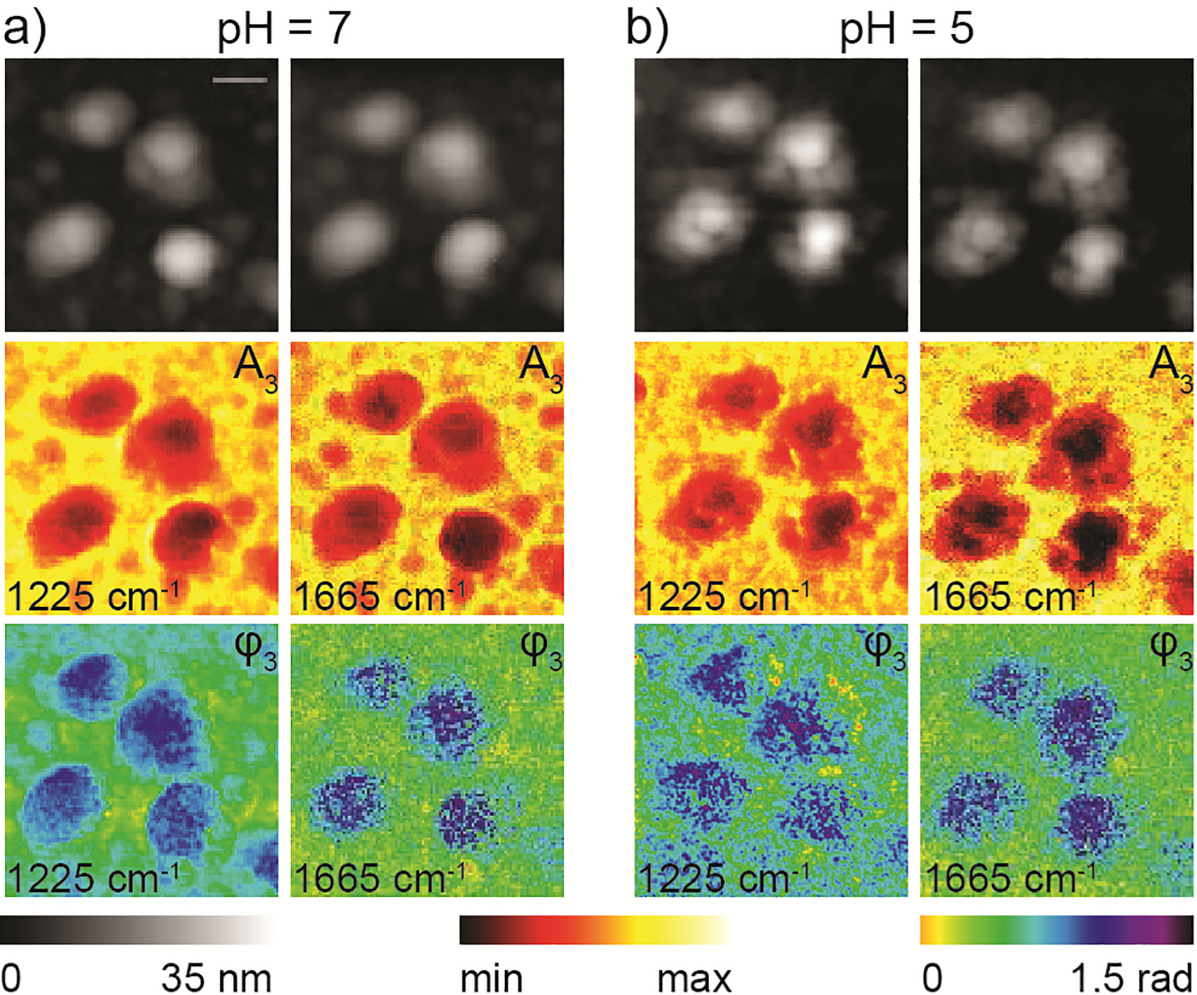
Spatial evolution of virus particles when acidity changed from pH 7 to pH 5. Topography (black & white), and near-field amplitude (A_3_) and phase (φ_3_) images of four virus particles at pH 7 (a) and pH 5 (b) taken at 1225 and 1665 cm^-1^. Scale bar 100 nm.

We first investigate the structural and spectral alterations in four virus particles as they are exposed to lower pH environment. Fig 2A and 2B show s-SNOM topography images (black & white), near-field amplitude (A_3_) and phase (φ_3_) images taken at two laser frequencies (1225 and 1665 cm^-1^ and pH = 5&7). Other even lower unphysiological pH exposures studies are given in the S2 Fig column-i (pH = 7) through column-v (pH = 2) with successively decreasing pH. Surprisingly, both the near-field spectral and the topography images show that as the viruses were exposed to increasing acidity they start to deform at few locations, visible from the edges towards the center of the particle (Fig 2A and 2B and S2 Fig). The membrane disruption progresses at multiple locations as the pH is further decreased (S2 Fig) and eventually the whole structure is fractured. The 1659 cm^-1^ image, which represents the amide I protein absorption (spectral assignments are discussed in detail below), shows a uniform protein distribution across the viruses. In contrast, the 1225 cm^-1^ image, that represents phosphate absorption, is fragmented, more and more so at lower pH. These differences in the 1665 and 1225 cm^-1^ images likely indicate disintegration of the lipid bilayer as well as RNA at low pH. This direct visualization of the effect of low pH on single virus particles clearly demonstrates viral membrane disruption in the absence of a host cell membrane, an intriguing observation that offers better insight into our current understanding of viral membrane fusion.

**Fig 3:**
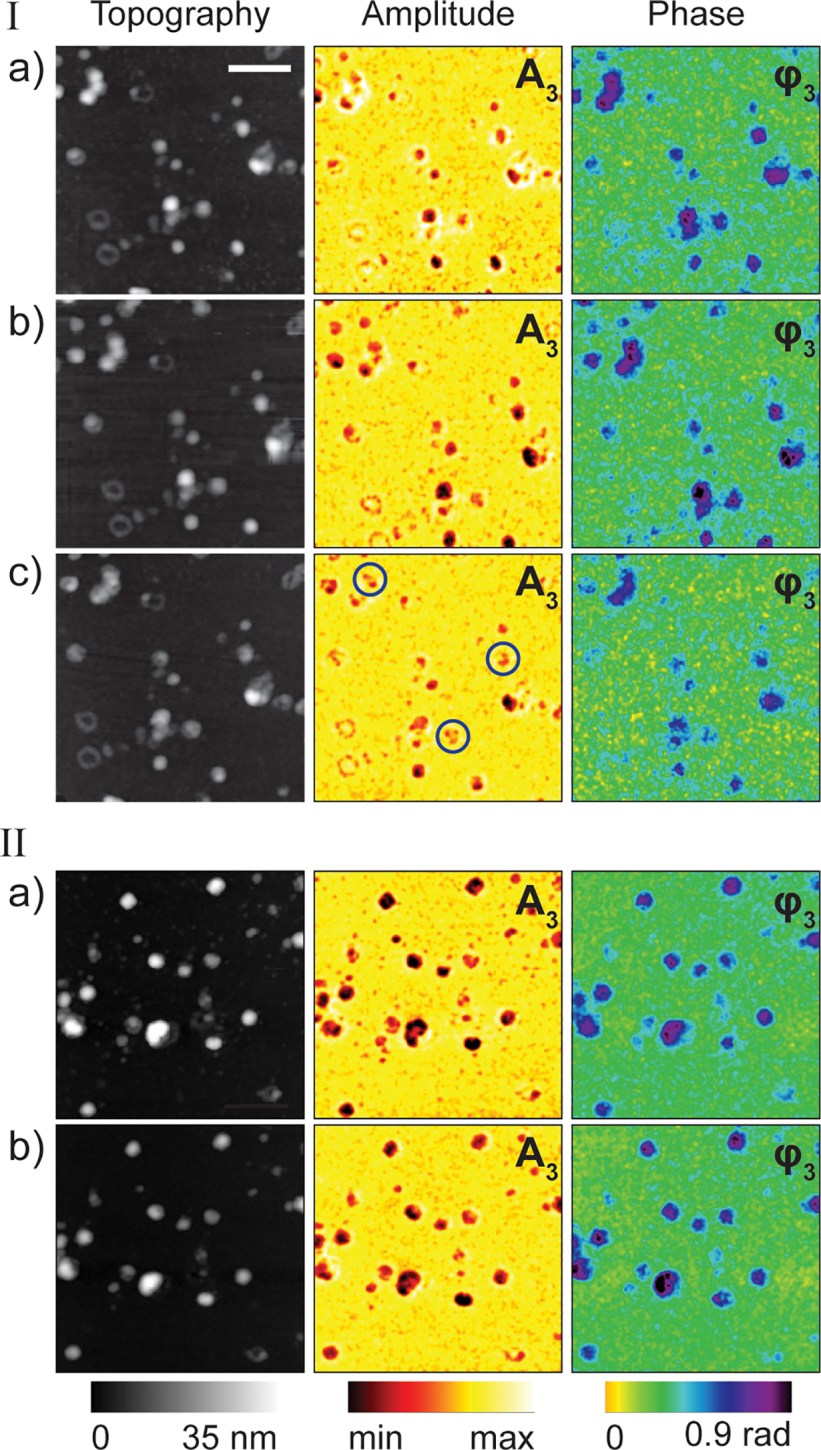
Topography, near-field amplitude (A_3_) and phase (φ_3_) images of several influenza virus particles i-(a), after compound 136 interaction (15 min) i-(b), followed by acid exposure i-(c). ii-(a-b) shows a different set of virus particles at neutral pH ii-(a) and ii-(b) shows the particles after acid exposure following compound 136 incubation (1 hr.). Scale bar 100 nm.

Prohibiting viral membrane disruption by antiviral compounds is an effective way of inhibiting viral infection. Our single virus nano-imaging technique allows assessment of the effectiveness of antivirals as we demonstrate below. Recently, we reported an antiviral compound (compound 136) that inhibits influenza virus fusion at picomolar concentrations in plaque reduction assays [12, 13]. Compound 136 was found to tightly bind to the viral envelope and inhibit influenza virus entry [12, 13]. Using our single virus particle imaging technique, we have characterized for the first time the effectiveness of this antiviral compound in stopping viral disruption during treatment with low pH solutions. This is achieved by following the same set of single viruses incubated with compound 136 and further exposed to low pH. In Fig 3 we show topography, near-field amplitude and phase images (taken at 1080 cm^-1^) of the same set of viruses before compound 136 treatment and neutral pH values (Fig 3A(I)), after incubation with 1 μM of compound 136 for 15 min (Fig 3B(I)) and then exposed to very high acidic solution (pH = 2, 15 min) (Fig 3C(I)). Careful examination of all the virus particles after incubation with compound 136 show that only 3 virus particles (indicated by black circles in the amplitude image in Fig 3C(I)) out of ~25 particles were fractured while others remained intact when they were exposed to low pH solution. We then increased the time of the virus incubation with 1 μM of compound 136 (to 1 hr) and exposed the particles to the same pH solution. The results in Fig 3A and 3B (II) show that all the particles remained intact after low pH exposure confirming the effectiveness of compound 136 in blocking viral membrane disruption induced by low pH.

**Fig 4:**
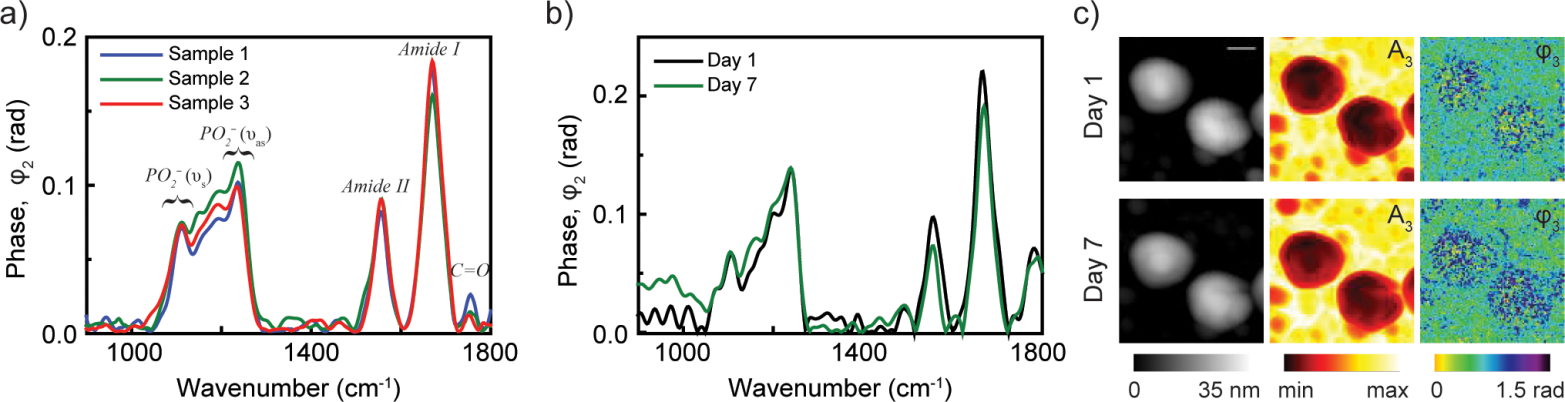
Nano-FTIR spectra of influenza virus particles from three different samples at neutral pH (a) and spectra from virus particles taken on day 1 and day 7 (b). Topography, near-field amplitude (A_3_) and phase (φ_3_) images of two influenza virus particles on day 1 and day 7 (c). Scale bar 100 nm.

To monitor the modification of chemical composition of a virus due to exposure to lower pH environment, we obtained infrared absorption spectra of single viruses before and after acid exposure. These single virus spectra before acid exposure are shown in Fig 4. A broadband light source integrated to s-SNOM (nano-FTIR) offers mapping of the vibrational fingerprints of a single virus at nanoscale spatial resolutions. Nano-FTIR takes advantage of the correlation of phase spectroscopic imaging to that of far-field absorption spectra and allow identification of materials through molecular fingerprint vibrational absorption spectra with a spatial resolution overcoming the diffraction limit by several orders of magnitude.[14, 15] The single virus spectrum is reproducible from sample to sample without being affected by sample preparation methods as shown in similar repeated spectra taken from different samples (Fig 4A). The viruses prepared by drop cast technique on silicon wafer are also stable with time both in topography and absorption spectra as shown in Fig 4B. As we describe below, the similarity of the spectra as shown in these controlled experiments with different samples performed at different days allow us to confidently assign the various peaks and follow their modification due to lower environmental pH.

In the IR spectra shown in Fig 4, the bands centered near 1658 and 1551 cm^-1^ are the well-known amide I and amide II absorption bands, respectively. These bands are predominantly due to the HA proteins. These bands may represent different protein secondary structures [16], or possibly contributions from multiple types of proteins. Amide I absorption at ~1629 cm^-1^ is often associated with β-sheet types of protein secondary structure that are abundant in the head of HA [16]. The lipids of influenza virus are in the 4 nm thick membrane envelope in which spikes of the HA proteins are inserted [17, 18]. The lipid membrane forms a key structural component of the virion providing an extraordinary biophysical stability to viruses and are associated directly with their infectivity. The peak centered near 1745 cm^-1^ in the IR spectra is well known to be associated with membrane lipids [19], in particular this peak is associated with lipid ester C = O absorption. Although this lipid C = O ester carbonyl absorption spectral region is free of other significant infrared-active modes, the exact peak position and bandwidth can vary from virus to virus with thermal history of the sample, the way in which the sample is prepared [20]. On the other hand, our ability to identify lipid ester C = O bands on a single virus is very useful since contour modification of the band can be related to structural modifications, hydration of the bilayer interface and the polarity and other properties of the local environment [21].

In the near-field IR spectra in Fig 4, a broad set of features spanning the 1290–1050 cm^-1^ region are observed, with the overall intensity of this set of features being less than the amide I absorption band. It is interesting to compare this observation to IR spectra of whole cells [19] which also generally display an amide I absorption band that is considerably more intense than absorption features in the 1290–1050 cm^-1^ region. Since the bands in the 1290–1050 cm^-1^ region are associated mainly with RNA/lipids, this implies that the ratio of RNA/lipids to protein derived from the IR spectra is less than 1.0 in influenza virus similar to that found in other biological cell types.

Lipids [22], carbohydrates [23] and RNA [24] all display absorption bands in the 1290–1050 cm^-1^ region [25]. As a result, this spectral range provides specific signature of the lipid bilayer and RNA which offers direct information on their chemical and structural modifications of enveloped viruses as surrounding environmental pH is varied. Antisymmetric and symmetric phosphate (PO_2_^-^) and RNA (PO_2_^-^) stretching vibrations are known to occur near 1230 and 1085 cm^-1^, respectively. We note that both of these asymmetric and symmetric stretching vibrations could contribute to the broad unresolved spectrum [26]. For both HCl treated and untreated viruses in the near-field spectra, it is difficult to disentangle the relative contribution of the lipid and RNA as well as other biomaterials. Simultaneously with spectroscopic information, s-SNOM also provides assessment of geometric changes of single virus particles either due to successively lower environmental pH or thermal/mechanical changes. As shown in S3 Fig, the virus particles in general decrease in height and increase in surface area at low pH.

**Fig 5:**
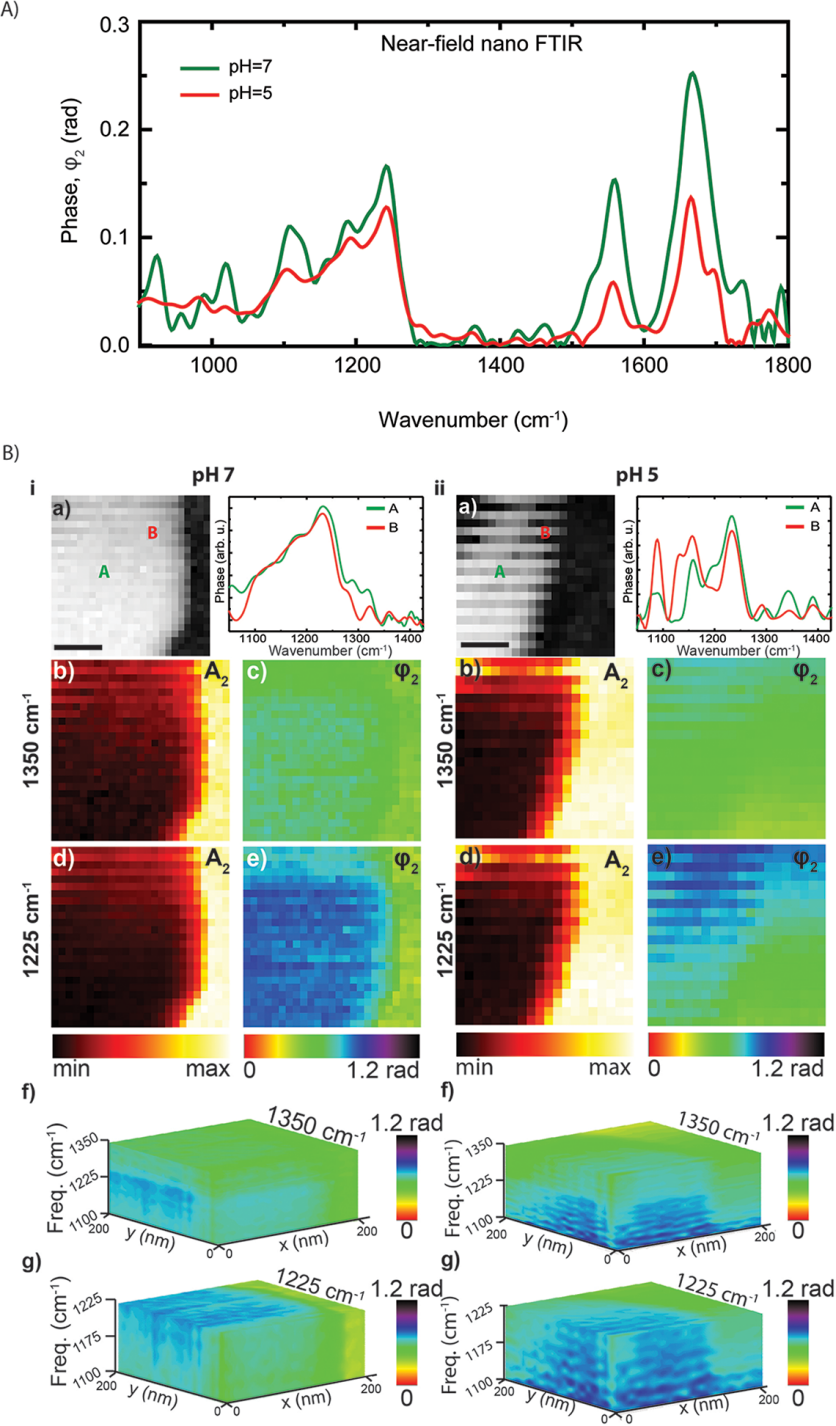
(A) Nano-FTIR spectra of influenza virus particles at neutral pH (green) and at pH 5 (red). Topography and hyperspectral images of a virus particle at pH 7. Topography, and spectra obtained at two different points on the particle (A and B) (a). Near-field amplitude, A_2_ (b and d) and phase φ_2_ (c and e) images at frequencies 1400 and 1225 cm^-1^ sliced from hyperspectral data. Scale bar 100 nm.

The nano-FTIR spectra on single virus particles at neutral pH (in green) and after exposure to acidic solution at pH 5 (in red) are plotted in Fig 5A. In the amide I and II regions, the near-field IR spectra before and after (green and red lines in Fig 5A, respectively) acid exposure, the general features are similar. This observation is consistent with the data in Fig 2A and 2B, which also show similar phase images at 1665 cm^-1^ both before and after acid treatment. However, both the amide I & II band intensities significantly reduced after acid treatment, which may indicate some, not unexpected, protein unfolding (of HA, M1, and NP proteins) upon treatment. The broad bands in the 1290–1050 cm^-1^ region do not seem to be significantly altered in these spectra. However, hyperspectral image taken [27] using IR broadband light source shows the broad spectrum in the 1290–1050 cm^-1^ region differs significantly from point to point on the virus surface (Fig 5). The hyperspectral images were acquired by scanning the probe tip over a selected surface area of the virus shown in the topography images (black-white) in Fig 5B. In this technique, an IR spectrum is taken at each pixel of the selected scanned area. This way, for each frequency value a 2D phase (or amplitude) map is created and these individual phase maps are stacked up as a function of frequency to result in a 3D near-field image shown in Fig 5B (f-g). From the 3D hyperspectral image, a single frequency 2D spectra or a corresponding 2D near-field image at any single frequency within the scanned range can be extracted. As an example, in Fig 5B next to each topography image, two IR phase spectra taken at two different locations, A and B on the particle directly extracted from the hyperspectral image are presented.

In this work, we have undertaken spectroscopy and imaging experiments on single influenza virus particles in order to investigate the chemical and structural modifications of the viral protein and lipid bilayer during various environmental pH variations and upon interaction with antiviral compounds. A remarkable finding of our work is that we directly show that if the environment pH is lowered, disruption of viral membrane could occur without the presence of a targeting membrane, contrary to the current viral fusion model, which requires virus binding to a host cell membrane for forming the fusion pore by fusion of two membranes to release the viral genome [28]. The fusion inhibitor compound 136 can effectively prevent the membrane disruption induced by low pH. Low pH may also cause uncoating of the M1 protein from the envelope when protons were introduced into the virion interior through the M2 channel. The overall structural changes of HA, membrane envelope and M1 protein may position the viral particle ready to release the genome. Our results are likely to be applicable to other enveloped viruses and may open unique opportunities for fundamental virology studies not offered by traditional ensemble methods.

## Methods

### Specimen preparation

To prepare the influenza virus Madin-Darby canine kidney (MDCK) cells (obtained from already-existing collection at GSU) were maintained with EMEM media complemented with 4% fetal bovine serum. Confluent MDCK cells were infected with influenza A virus (STRAIN X-31) at a MOI of 1.0 in EMEM media containing 2 μg/mL TPCK-treated trypsin, and the infected cells were incubated at 37°C, 5% CO_2_. The media were collected after 48 hours post infection. After clarification to remove cell debris, the virus was pelleted through a 33% sucrose cushion at 85,000 x g. The virus pellet was resuspended in PBS and pelleted again through a 30% glycerol cushion at 15,000 x g. The virus pellet was resuspended in PBS and stored at 4°C before usage. The virus particles are then drop casted on pre-cleaned Si wafer for s-SNOM experiments. After sitting for 15 min, unbound particles were washed off with water. For changes of condition, the particle deposited wafer was incubated with water at various pH values adjusted with a stock solution of 1M HCL, or 1 μM of compound 136 in 1% DMSO.

### Near-field s-SNOM nanospectroscopy

**Fig 6:**
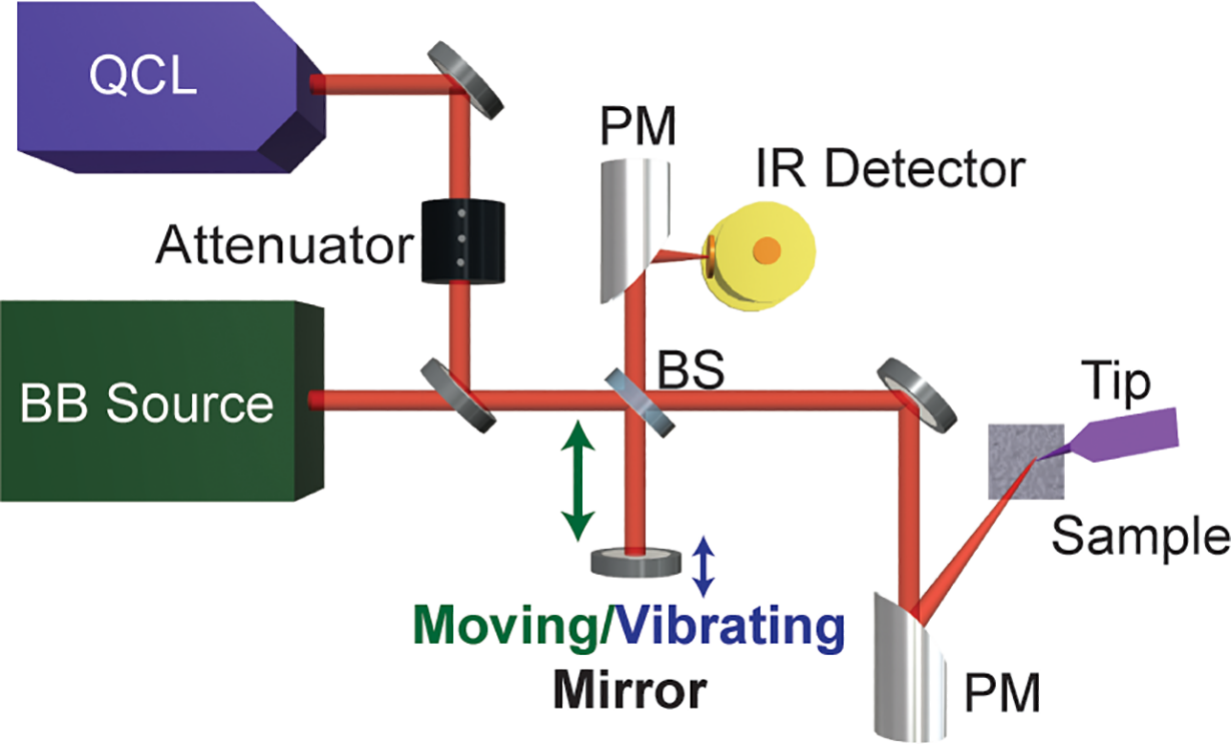
Schematic of s-SNOM nanoimaging and nano-FTIR setup.

Fig 6 show the scattering type scanning near-field optical microscope (s-SNOM) nanoimaging setup with nano-FTIR module integrated (http://www.neaspec.com/). The probe tip in s-SNOM functions as a local probe via scattering of the nanolocalized optical fields from a probe tip either at single frequency or using broadband light sources covering wide IR finger print frequency range.[8–12] The probe tip is coated with PtIr and oscillates at resonance frequency of *f*~280 kHz. For single frequency nanoimaging, it is irradiated by focused quantum cascade lasers (QCLs) (http://www.daylightsolutions.com/) at 45° with respect to the sample surface using a parabolic mirror (PM). The scattered signal from the tip-sample interface region is demodulated at high harmonics of the tip resonance frequency (*nf*, n>1) and detected by phase-modulation (pseudo-heterodyne) interferometry using a beam splitter (BS) and a vibrating mirror. s-SNOM nanoimaging results in simultaneous topography, IR amplitude and phase images that offer complementary information. The heights of the viruses were measured from the topography image. The near-field phase, being directly proportional to the imaginary part of the permittivity, represents vibrational absorption of a sample [8, 9]. For obtaining nano-FTIR spectra, a broadband (BB) mid-infrared laser source that coupled to the s-SNOM was used to illuminate the AFM tip. The backscattered light was analyzed using the Michelson interferometric configuration that consists a moving mirror to create a reference light beam, as shown in Fig 6.

## Supporting information

S1 Fig High resolution s-SNOM topography and amplitude images showing outer viral envelop and the inner structure of a single influenza virus. (a) Topography of a virus particle and (b) 3rd harmonic near-field amplitude image taken at 1045 cm-1 showing contrast difference between the outer viral envelop and the inner structure of the virus. (c) Topography and amplitude line profiles. Scale bar is 100 nm.
(PDF)

S2 Fig Spatial evolution of virus particles at progressively more acidic pH. Topography (black & white), scale bar 100 nm and near-field amplitude (A3) and phase (φ3) images of four virus particles at progressively lower pH (left to right). Spectral images taken at (a) 1225 cm-1. The columns i-v in the topography represent successively decreasing pH from neutral (i) to pH 2 (v).
(PDF)

S3 Fig Geometric changes of virus particles due to acid treatments. Geometric, height (black points) and surface area (blue points), evolution plot of influenza x-31 virus particles as a function of decreasing pH from neutral (i) to pH 2 (v).
(PDF)
