# Supplementary material for: Probing structural changes in single enveloped virus particles using nano-infrared spectroscopic imaging
Source: PLoS One. 2018 Jun 12;13(6):e0199112. doi: 10.1371/journal.pone.0199112 (PMC5997350; doi:10.1371/journal.pone.0199112)
Supplement: S1 Fig — (a) Topography of a virus particle and (b) 3rd harmonic near-field amplitude image taken at 1045 cm-1 showing contrast difference between the outer viral envelop and the inner structure of the virus. (c) Topography and amplitude line profiles. Scale bar is 100 nm. (PDF) [file pone.0199112.s001.pdf]

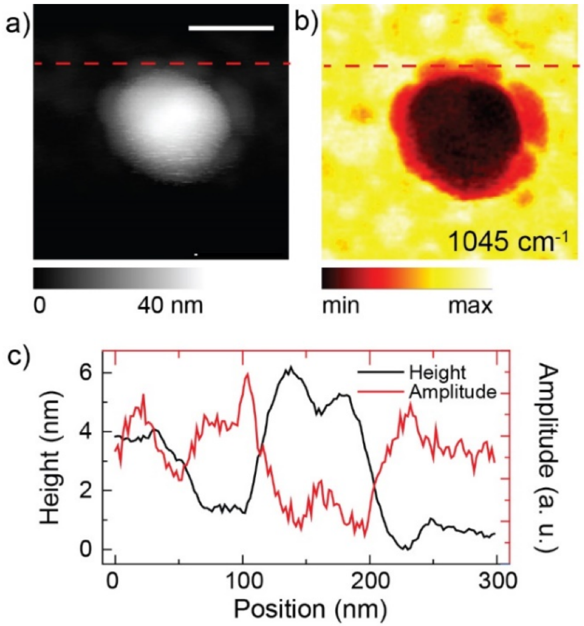

Figure S1. High resolution s-SNOM topography and amplitude images showing outer viral envelop and the inner structure of a single influenza virus. (a) Topography of a virus particle and (b) 3rd harmonic near-field amplitude image taken at 1045 cm^{-1} showing contrast difference between the outer viral HA protein and the inner structure of the virus. (c) Topography and amplitude line profiles. Scale bar is 100 nm.
