# Supplementary material for: Probing structural changes in single enveloped virus particles using nano-infrared spectroscopic imaging
Source: PLoS One. 2018 Jun 12;13(6):e0199112. doi: 10.1371/journal.pone.0199112 (PMC5997350; doi:10.1371/journal.pone.0199112)
Supplement: S2 Fig — Topography (black & white), scale bar 100 nm and near-field amplitude (A3) and phase (φ3) images of four virus particles at progressively lower pH (left to right). Spectral images taken at (a) 1225 cm-1. The columns i-v in the topography represent successively decreasing pH from neutral (i) to pH 2 (v). (PDF) [file pone.0199112.s002.pdf]

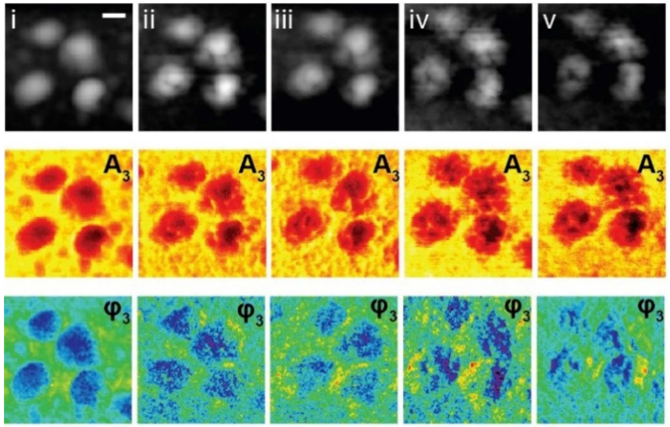

Figure S2. Spatial evolution of virus particles at progressively more acidic pH. Topography (black & white), scale bar 100 nm and near-field amplitude (A_3) and phase (ϕ_3) images of four virus particles at progressively lower pH (left to right). Spectral images taken at (a) 1225 cm^{-1}. The columns i-v in the topography represent successively decreasing pH from neutral (i) to pH 2 (v).
