# Supplementary material for: Probing structural changes in single enveloped virus particles using nano-infrared spectroscopic imaging
Source: PLoS One. 2018 Jun 12;13(6):e0199112. doi: 10.1371/journal.pone.0199112 (PMC5997350; doi:10.1371/journal.pone.0199112)
Supplement: S3 Fig — Geometric, height (black points) and surface area (blue points), evolution plot of influenza x-31 virus particles as a function of decreasing pH from neutral (i) to pH 2 (v). (PDF) [file pone.0199112.s003.pdf]

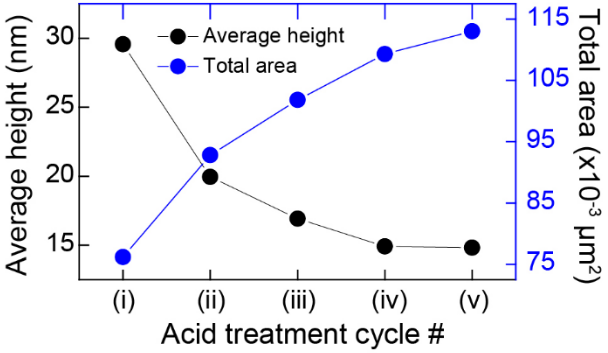

Figure S3. Geometric changes of virus particles due to acid treatments. Geometric, height (black points) and surface area (blue points), evolution plot of influenza x-31 virus particles as a function of decreasing pH from neutral (i) to pH 2 (v).
